# Profiling of subgingival plaque biofilm microbiota in adolescents after completion of orthodontic therapy

**DOI:** 10.1371/journal.pone.0171550

**Published:** 2017-02-03

**Authors:** Shuang Pan, Yi Liu, Li Zhang, Shuxiang Li, Yujie Zhang, Jianwei Liu, Chunling Wang, Shuiqing Xiao

**Affiliations:** 1 Department of Orthodontics, School of Stomatology, Shandong University, Jinan, China; 2 Department of Orthodontics, Jinan Stomatological Hospital, Jinan, China; 3 Pediatric Research Institute, Qilu Children’s Hospital of Shandong University, Jinan, China; 4 Department of Stomatology, Hospital of Zhangqiu, Jinan, China; 5 Department of Oral Medicine, Shandong Medical College, Jinan, China; University of the Pacific, UNITED STATES

## Abstract

**Background:**

Fixed orthodontic treatment is the most common method for malocclusion but has the potential risk of periodontal complication with unclear outcomes of whether microbiologic and clinical changes could be reversible in adolescents after orthodontic therapy.

**Methods:**

Twenty adolescents with orthodontic treatment were enrolled in the study as the case group at end of the therapy, while 19 periodontally healthy adolescents were involved in the control group. At baseline (T_0_), clinical parameters including gingival index, probing depth and sulcus bleeding index were tested, and subgingival plaque samples were collected from the lower incisors. The counts of *A*. *actinomycetemcomitans*, *P*. *gingivalis*, *P*. *intermedia*, *T*. *forsythia* and total bacteria were determined by real-time PCR. All parameters were reassessed after 1 month (T_1_) and 3 months (T_2_) in the case group and compared with that of the controls.

**Results:**

At baseline (T_0_), clinical parameters (including GI, PD, SBI) of the test sites in the case group were significantly higher than that of the control group (*P*<0.05 or *P*<0.01). At 3 months (T_2_), no differences were noticed in GI and SBI between two groups. The prevalence and counts of periodontopathogens tend to be normal (*P*>0.05), while PD and the amount of *P*.*intermedia* were still significantly higher compared with that of the control group (*P*<0.05 or *P*<0.01).

**Conclusion:**

After removal of appliances, the periodontal changes induced by orthodontic therapy are only partially reversible at 3 months after removal.

## Introduction

Fixed orthodontic therapy is an effective and common method for treating malocclusions in contemporary orthodontics. One of the common side effects during orthodontic therapy is periodontal complication including gingivitis and periodontitis. It is still unclear whether the periodontal changes in orthodontic therapy could be reversible after the removal of appliances. Most studies indicated that gingival changes were only temporary and could be reversible [1–5], while a few of researches reported a significantly clinical attachment loss during orthodontic therapy [6–8]. A prospective study discovered that orthodontic accessories had a negative impact on periodontal parameters, moreover these changes were only partially reversible post-therapy [7].

It has been recognized that anaerobic microorganisms in the subgingival plaque are the key etiologic factors in the initiation and progression of gingivitis and periodontitis [9]. Recent advancements in the periodontal research field supported the theory that periodontal diseases are resulted from a rupture of the dynamic balance between the relative abundance of periodontopathogens and host defense system [10]. Positive associations between periodontal diseases and several pathogens have been reported, including *A*. *actinomycetemcomitans*, *P*. *gingivalis*, *P*. *intermedia*, and *T*. *forsythia* [11–13]. The previous research revealed that *T*. *forsythia* and *P*. *gingivalis*, have been categorized as the “read complex” species, which are related to the severity of periodontitis, while *A*. *actinomycetemcomitans* and *P*. *intermedia* are categorized as secondary harmful species involved in periodontitis [14–18]. Additionally, current evidence suggested orthodontic appliances could alter the equilibrium of the microorganism ecosystem, and increased the potential for pathogenicity within the microbial ecosystem [19, 20]. Our previous study demonstrated that the levels of *P*. *gingivalis* and *F*. *nucleatum* were obviously elevated after placement of orthodontic appliances [21, 22]. Therefore, it is crucial to understand the composition and changes of periodontopathogens during orthodontic therapy in order to avoid potentially irreversible injuries caused by orthodontic appliances.

In this study, we quantify subgingival pathogens of *A*. *actinomycetemcomitans*, *P*. *gingivalis*, *P*. *intermedia*, *T*. *forsythia* and total bacteria with the 16S rRNA based real-time PCR and tested the clinical parameters in adolescents among different time points after orthodontic treatment to assess whether the microbial and periodontal parameters could be reversible after removal of orthodontic appliances.

## Materials and methods

### Ethics statement

This study was approved by the Ethics Committee of Jinan Stomatological Hospital and School of Stomatology Shandong University. Written informed consents were obtained from the patients or their parents. The patients’ information was anonymized during or after data collection.

### Determination of the sample size

We firstly searched for eligible reports published between 30^th^, October 2005 and 30^th^, October 2015 in PubMed with the following terms: ‘gingivitis’/ or ‘periodontal pathogens’/ or ‘real-time PCR’/ or ‘quantitative PCR’ in various combinations. We excluded reports without concrete data, as well as trials without pathogens’ absolute quantitation. Six trials with 1010 subjects were included. The average value of mean and standard deviation (SD) of four pathogens (*A*. *actinomycetemcomitans*, *P*. *gingivalis*, *P*. *intermedia*, and *T*. *forsythia*) in gingivitis /or mild periodontitis group and health control group were calculated and transformed in logarithmic form (S4 Table).

Sample size was determined by PASS 2008 (Power Analysis and Sample size, V08.0.3). A two-sided two sample *t* test was used with the following assumptions: α of 0.05, power of 80%, differences in mean amount and standard deviation of periodontopathogens between case group and control group using information from previous studies[23–28] (S1 Table). Finally, a minimum of 18 subjects in each group were predicted to detect the difference in the counts of pathogens.

### Subjects

Subjects of both case and control groups were recruited from Department of Orthodontics, Jinan Stomatological Hospital during June 2015- October 2015. The case group consisted of 20 adolescent subjects (8 males and 12 females, mean age 14.42±0.86 years) who underwent fixed orthodontic therapy for 17.9 months to 22.9 months(mean timing 20.07±1.57 months) from July 2013 to September 2015, without clinical signs of gingivitis or periodontitis before orthodontic therapy. The control group was comprised of 19 periodontally healthy individuals without receiving orthodontic therapy (9 males and 10 females, mean age 14.24±0.62 years, S2 Table) matched for age and sex, according to the following criteria: ⑴ no smoking; ⑵ no known systemic disease; ⑶ no alveolar bone loss visible on X-ray; ⑷ no fixed restorations or removal dentures; ⑸ no use of antibiotics within 3 months before the study; ⑹ no periodontal therapy within the previous 6 months. Before the study, all participants were received a standardized oral hygiene instruction by the same orthodontist. All orthodontic patients were placed the maxillary and mandibular Hawley removable retainers after removal of the fixed appliance.

### Clinical measurement

For both groups, clinical parameters including gingival index (GI, S3 Table), probing pocket depth (PD), and sulcus bleeding index (SBI, S4 Table) were assessed at the teeth of 31, 32, 41 and 42[29, 30]. The score for each participant was the mean measurements of the four teeth. In the case group, measurements were made at three time points: Baseline T_0_ = before appliances removal, T_1_ = one month after removal, and T_2_ = three months after removal.

Periodontal examination and sample collection were performed by the same orthodontist. Probing pocket depth (PD) which was measured in millimeters at three sites (mesiobuccal, midbuccal, and distalbuccal) per tooth by using a periodontal probe (Hu-Friedy, Chicago, America), is the distance between the gingival margin and the bottom of periodontal pocket or gingival sulucs.

### Microbial sampling

The participants were asked to refrain from oral hygiene measures, eating and drinking for two hours before sample collection. Subgingival microbial samples were collected from the four teeth of 31, 32, 41 and 42. Prior to sampling, supragingival plaque was carefully removed and the sampling sites were isolated with sterile cotton rolls and gentle air drying. A sterile filter paper strip (ISO 30) was softly inserted into each packet for 30 seconds until a minimum of resistance [31]. Because real-time PCR often failed to detect the pathogens in the control sample tube with only one sterile filter paper, we referred to previous documents [5, 23, 32–35] and finally determined to place four paper points of each subject into a sterile microcentrifuge tube containing 0.5 ml of PBS to perform the PCR. The tubes were stored at -20°C until analyzed.

We selected the lower incisors as sampling sites because: (1) it’s been reported that both upper posterior teeth and anterior teeth had higher prevalence of periodontopathogens and could be the representative for study of oral microbiology [1], moreover, it is relatively convenient and easy to avoid inclusion of saliva when collecting samples from anterior teeth; (2) Gingival hyperplasia often occurs at the posterior and canine teeth in orthodontic patients with teeth extraction, which is attributed to teeth movement and orthodontic force rather than the gingivitis, so the clinical periodontal parameters and microbial parameters might be inconsistent on this occasion. (3) Referred to previous studies [5, 24] that collected subgingival plaque from anterior teeth; (4) Ramfjord teeth consist of the tooth 16, 21, 24, 36, 41, 44. In patients with moderate to severe dental crowding malocclusion, the tooth 24 and 44 are usually extracted, and gingival hyperplasia attributed to the teeth movement and orthodontic force often occurs at the tooth 16 and 36. Therefore, we selected lower incisors as sampling sites.

### Reference strains

Four reference strains of anaerobic bacteria used in this study included *P*. *gingivalis* (ATCC33277), *A*. *actinomycetemcomitans* (ATCC 700685), *P*. *intermedia* (ATCC 25611), and *T*. *forsythia* (ATCC 43037). The first three of *P*. *gingivalis*, *A*. *actinomycetemcomitans*, *P*. *intermedia* were obtained from The State Key Laboratory of Oral Diseases, Sichuan University (Chengdu, China), and the last one (*T*. *forsythia*) was purchased from the American Type Culture Collection (ATCC, Manassas, VA). All anaerobes organisms were cultured in an anaerobic chamber in the required growth media with vitamin K1, L-cystein, and hemin at 37°C for about 7–10 days.

### DNA extraction

Genomic DNA was extracted from the subgingival plaque samples and bacterial strains, using the TIANamp Micro DNA Kit (Tiangen, Beijing, China), according to the manufacturer’s instructions. DNA concentrations were determined with a NanoDrop Spectrophotometer (Thermo Scientific, CA, US).

### Real-time PCR primers

The species-specific primers of the *A*. *actinomycetemcomitans* and *T*. *forsythia* designed in the 16S rRNA gene were described previously [25] and shown in Table 1, the primers of *P*. *gingivalis* and *P*. *intermedia* were designed by Beijing Genepool Technologies Company (Beijing, China). An universal primer pair based on highly conserved region of the 16S rRNA gene was used to detect all bacterial species in the samples according to the description of Maeda et al. [36] (Table 1). All primers were checked for possible cross-hybridization with bacterial genes using the database similarity search program BLAST.

**Table 1:** Target bacteria and their species-specific primers used in the present study.

Species	DNA Sequence (5'-3')	Product (bp)	Annealing temp (°C)	References
*Aggregatibacter actinomycetemcomitans*	TGTGCCTTAGGGAGCTTTGAGACA	106	59	[25]
	GCAACAAAGGATAAGGGTTGCGCT			
*Porphyromonas gingivalis*	GGAATAACGGGCGATACGA	155	59	
	CACCGCTGACTTACCGAACA			
*Prevotella intermedia*	GCCTAATACCCGATGTTGTCC	237	59	
	CACCGCTGACTTACCGAACA			
*Tannerella forsythia*	ACACCTCCTTTCTGGAGCAGTCTT	138	61	[25]
	AACCGAGACATCCCAGCTTCCTTT			
Universal	GTGSTGCAYGGYTGTCGTCA	146	60	[26]
	ACGTCRTCCMCACCTTCCTC			

### Generation of recombinant positive controls and standard curve

We used plasmids and standard curve to calibrate the counts of pathogens. PCR quantification standards were prepared using recombinant plasmids containing the amplified 16S rRNA region of each target bacterium. The recombinant plasmids containing the amplified region of each target bacterium were constructed and synthesized by Beijing Genepool Technologies Company (Beijing, China) to be as the positive controls. The plasmids DNA were quantified by spectrophotometric measurement of the absorbance at 260 nm after sequencing validation. The copy numbers of positive controls of the four individual bacteria were calculated based on molecular mass. Standard curve were established with the positive plasmid standards by placing them in serial 10-fold dilutions, ranging from 10 to 10^6^ DNA copies for *P*. *gingivalis*, *P*. *intermedia*, *A*. *actinomycetemcomitans*, *T*. *forsythia* and total bacteria. The standard curve was generated as a plot between the cycle number at the crossing point and the initial plasmid DNA copies. Using the standard curve, absolute quantity of each target species was calculated as the logarithm of DNA copies by ABI 7500 Software v2.0.1.

### Quantitative real-time PCR assay

All clinical samples and plasmid standards were run in triplicate following manufacturer recommendation of the ABI7500 Fast qPCR System (Applied Biosystems, Inc Foster, CA). The qPCR was performed according to the conditions (Table 1). Quantification of each target gene copy numbers was obtained by comparison with standard curves.

### Statistical analysis

The data were analyzed by using the SPSS statistical software (version 20.0, SPSS, Chicago, III). The differences in sex and age of both groups were analyzed using the Fisher exact test and the Independent t-test.

The log_10_ transformation was applied to the microbiologic data for normalizing the distribution and stabilizing the variance. The clinical periodontal parameters and microbiologic parameters were described with medians, 25th percentile (Q1), and 75th percentile (Q3).

The Mann-Whitney U test and the Wilcoxon signed rank test were used to detect significant intergroup and intragroup differences regarding clinical periodontal parameters and microbiologic parameters. The Fisher exact test was used for pairwise comparisons of the frequencies of periodontopathogens. The significance for all statistical tests was predetermined at *P*<0.05.

## Results

There was no statistical difference in the sex and age of the members of the two groups (*P*>0.05, S1 Table).

### Specificity of the real-time PCR primers

To determine the specificity of the qPCR primers, the recombinant plasmid DNA of the four target bacteria were tested with the five pairs of primers individually, displaying that all species-specific primers could only amplify the correct target DNA and no cross-reactivity was observed, while the universal primes for all the bacteria could amplify the four bacteria of *P*. *gingivalis*, *P*. *intermedia*, *A*. *actinomycetemcomitans*, *T*. *forsythia*.

### Prevalence of the four bacteria

**Fig 1:**
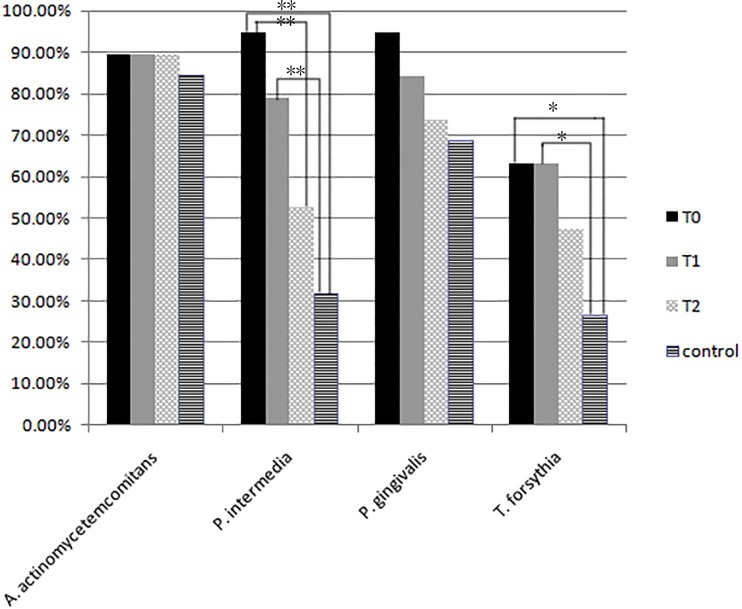
Prevalence of subgingival microorganisms in case and control group. The Fisher exact test was used for pairwise comparisons of the frequencies of periodontopathogens (* *P< 0*.*05*, **P<0.*0*).

The frequencies of periodontopathogens were showed in Fig 1. At baseline, *A*. *actinomycetemcomitans* and *P*. *gingivalis* was detected in 89.50% and 94.73% of cases, respectively, with no difference from that of control group (*P*>0.05). However, P. *intermedia* was showed a statistical difference between the case group and control group with positive rate of 94.73% in cases and 31.58% in controls (*P*<0.01); *T*. *forsythia* was detected in 63.16% of case group and 26.31% of control group, with a significant difference (P<0.05).

At T2, the frequencies of pathogens in the case group were 89.50% for *A*. *actinomycetemcomitans*, 73.68% for *P*. *gingivalis*, 52.63% for *P*. *intermedia*, 47.39% for *T*. *forsythia*, and no significant difference between the two groups (*P*>0.05). There was no significant change for *A*. *actinomycetemcomitans*, *P*. *gingivalis* and *T*. *forsythia* over time from T_0_ to T_2_ (*P*>0.05), while the significant decreases were noticed for *P*. *intermedia* (*P*<0.01) from T_0_ to T_2_.

### Detection of clinical specimens

The absolute bacterial counts (log_10_ transformed from copy numbers) of *P*. *gingivalis*, *P*. *intermedia*, *A*. *actinomycetemcomitans*, *T*. *forsythia* and total bacteria were quantified in both groups of clinical specimens, containing 19 samples of case group and 19 samples of control group. The proportions of the four bacterial species were calculated by using the absolute bacterial counts of each and total bacteria (see Table 2).

**Table 2:** Bacterial loads in subgingival plaque from the orthodontic patients and periontally healthy subjects.

	Case group									Control group		
	T_0_			T_1_			T_2_			T_0_		
	Median	25th percentile	75th percentile	Median	25th percentile	75th percentile	Median	25th percentile	75th percentile	Median	25th percentile	75th percentile
Aa (log_10_)	3.34	2.79	3.93	2.95	2.48	3.17	2.83	2.46	3.46	3.33	2.90	3.99
Pi (log_10_)	3.99 ^††^	1.81	5.32	3.65 ^††^	1.89	4.43	1.77 **^†^	0.00	2.91	0.00	0.00	1.38
Pg (log_10_)	3.48 ^†^	1.70	5.16	1.72 *	1.64	5.02	1.68 **	1.60	2.38	1.70	1.68	1.79
Tf (log_10_)	3.34^††^	0.00	3.80	3.01 ^††^	0.00	3.59	0.00 *	0.00	3.20	0.00	0.00	1.29
Total (log_10_)	5.82	5.65	6.30	5.64 **	5.10	5.82	5.29 **	4.77	5.62	5.60	5.13	5.86
Aa/total (%)	0.26	0.18	0.87	0.30	0.06	1.48	0.42	0.22	1.23	0.75	0.11	2.62
Pi/total (%)	1.21 ^††^	0.02	10.33	1.10 ^††^	0.04	4.94	0.02 *	0.00	0.11	0.00	0.00	0.01
Pg/total (%)	0.39 ^†^	0.01	12. 82	0.08 ^†^	0.02	15.85	0.06	0.01	1.21	0.01	0.01	0.04
Tf/total (%)	0.13 ^††^	0.00	0.53	0.12 ^††^	0.00	0.59	0.00	0.00	0.67	0.00	0.00	0.00

The absolute bacterial counts were log10 transformed. The proportions of the four bacterial species were calculated by using the absolute bacterial counts of each species and total bacteria.

Wilcoxon signed rank test indicated significant difference in comparison with T_0_

* *P*<0.05.

** *P*<0.01.

Mann-Whitney U test indicated significant difference in comparison with the control group.

† *P*<0.05.

†† *P*<0.01.

The results showed that at baseline, the median count and proportion were 3.49 and 0.39% for *P*. *gingivalis*, 3.99 and 1.21% for *P*. *intermedia*, 3.34 and 0.13% for *T*. *forsythia* in case group, which was found at higher level compared with the healthy controls (*P*. *gingivalis P*<0.05, *P*. *intermedia P*<0.01, *T*. *forsythia P*<0.01). At T_2_, the median count and proportion of cases decreased to 1.72 and 0.08% for *P*. *gingivalis*, 3.65 and 1.14% for *P*. *intermedia*, 3.01 and 0.12% for *T*. *forsythia*, which was statistically different from that at baseline (*P*. *gingivalis P*<0.01, *P*. *intermedia P*<0.01, *T*. *forsythia P*<0.05). The count and proportion of *A*. *actinomycetemcomitans* in case group showed no significant change over time from T_0_ to T_2_, and there was no difference between two groups of case and control (*P*>0.05). Compared with control group, no significant differences were found in the amount of total bacteria (*P*>0.05). At T_2_, there was no significant difference in the counts and proportions between two groups for the pathogens, except for the count of *P*. *intermedia* (*P*<0.05).

### Evaluation of clinical parameters

We evaluate the clinical parameters. At baseline (T_0_), all clinical parameters, including GI, PD, SBI in case group were found significantly higher than those in control group (*P*<0.01, Table 3). After removing appliances, GI, PD and SBI significantly decreased from T_0_ to T_2_ (*P*<0.01). At T_2_, no significant differences were observed concerning the GI and SBI between two groups (*P*>0.05). However, PD was still higher in case group than that in control group (*P*<0.01).

**Table 3:** Clinical parameters from the orthodontic patients and periodontally healthy subjects.

	Case group									Control group		
	T_0_			T_1_			T_2_			T_0_		
	Median	25th percentile	75th percentile	Median	25th percentile	75th percentile	Median	25th percentile	75th percentile	Median	25th percentile	75th percentile
GI	1.50 ^††^	1.00	1.50	1.00 **^††^	1.00	1.25	0.00 **	0.00	0.05	0.00	0.00	0.00
PD(mm)	2.50 ^††^	2.00	3.00	2.00 **^††^	2.00	2.50	1.50 **^††^	1.50	2.00	1.50	1.50	1.50
SBI	1.00 ^††^	1.00	2.00	0.00 **	0.00	0.00	0.00 **	0.00	0.00	0.00	0.00	0.00

GI, gingival index; SBI, sulcus bleeding index; PD, probe depth.

Wilcoxon signed rank test indicated significant difference in comparison with T_0_.

* *P*<0.05.

** *P*<0.01.

Mann-Whitney U test indicated significant difference in comparison with the control group.

† *P*<0.05.

†† *P*<0.01.

## Discussion

The previous studies on the periodontal pathogens of orthodontic patients mostly focused on the prevalence of pathogens using regular PCR with very little information on the exact quantitative variation. However, the main difference between the periodontal disease and healthy status was predicted to be the amount of pathogens rather than prevalence according to previous research [32]. To our best knowledge, this is the first report regarding the absolute quantification of various periodontopathogens and amount of total bacteria detected by real-time PCR in subgingival samples after appliances removal. These results provide important information toward profiling subgingival plaque microbiota from adolescents after completion of orthodontic therapy.

It is known there is an obvious improvement of clinical parameters after appliances removal [1,20,37]. Van Gastel et al. reported that probing depth (PD) and bleeding on probing (BOP) reduced significantly after appliances removal, but remained obviously higher than that at pre-therapy [37]. Consistent with this study, our data showed, 3 months after removal, PD was still significantly higher compared with the healthy controls. The increased PD recorded may be ascribed to fibrotic changes in gingival connective tissues. Once fibrotic changes have taken place, removing the irritating factors, such as bacterial plaque, archwires, and brackets, could not return the gingival tissues to their normal physiologic contour [31, 38].

Several studies showed that the frequencies and counts of periodontopathogens significantly decreased after appliances removal [1–4, 20, 26]. Kim stated that *P*.*gingivalis*, *P*. *intermedia* and *T*. *forsythia* dramatically decreased immediately after appliances removal, but *A*. *actinomycetemcomitans* remained unchanged [26]. Similarly, our study presented that there was no difference in the prevalence between two groups 3 months after therapy. Furthermore, the counts and proportions of *P*. *gingivalis*, *P*. *intermedia* and *T*. *forsythia* decreased obviously, the count of *A*. *actinomycetemcomitans* did not change over time. This is due to the fact that the gingival enlargement induced by orthodontic appliances might provide a favorable environment for the colonization and maturation of anaerobic bacteria, and favors a qualitative shift from a predominance of aerobic bacteria to more putative anaerobic periodontal pathogens [39, 40]. Therefore, removing the orthodontic appliances eliminates their plaque-retentive effect, which might make practicing good oral hygiene easier [39]. Additionally, this discrepancy of nonsignificant difference in the count of *A*. *actinomycetemcomitans* as opposed to significant differences in the counts of *P*. *gingivalis*, *P*. *intermedia* and *T*. *forsythia* may be ascribed to the fact that *A*. *actinomycetemcomitans* is a gram-negative facultative anaerobe, while *P*. *gingivalis*, *T*. *forsythia* and *P*. *intermedia* are obligate anaerobes, the growth of latter are easily to be stimulated by the ecologic environment induced by gingival enlargement.

Interestingly, in our study, *P*. *intermedia* were still significantly more abundant in case group compared with healthy controls at T_2_. It is widely accepted that the presence of some oral anaerobes might be associated with the hormonal level, especially during the puberty [41]. The previous research revealed that there is a higher incidence of *P*. *intermedia* in the subgingival microflora in puberty [42]. This may explain the count of *P*. *intermedia* was still higher than that of control group, whereas the counts of *P*. *gingivalis* and *T*. *forsythia* returned to be normal after improvement of oral hygiene. Furthermore, at T_2_, PD in case group was still higher than control group, which provide a favorable environment for the colonization and maturation of *P*. *intermedia*. Therefore, it is reasonable to evaluate the count of *P*. *intermedia* in subgingival plaque from adolescents after orthodontic therapy for more than 3 months. If no further changes occur, it would be justifiable to carry out periodontal treatment 3 months after therapy to reduce PD and the count of *P*. *intermedia*.

At baseline, we also observed there was no significant difference in the count of total bacteria between two groups, whereas the counts and proportions of *P*. *gingivalis*, *T*. *forsythia* and *P*. *intermedia* were higher compared with healthy controls. This result probably supports the hypothesis that periodontal diseases may be caused by some specific microorganisms, rather than excessive accumulation of microorganisms [43]. Armitage stated that periodontally healthy subjects carry putative periodontal pathogens as part of the normal oral plaque because these bacteria are detected at low numbers in periodontally healthy individuals [44]. These microorganisms may be opportunistic pathogens, the levels of which increase to a critical threshold to induce periodontal tissues destruction. Accordingly, we observed that there was no significant difference in the prevalence of *P*. *gingivalis* between two groups at baseline, while the count of *P*. *gingivalis* was obviously higher in case groups, in accordance with a previous study [23]. This implies that an increased number of periodotopathogens, rather than frequency, might be an important determinant in the development of periodontal inflammation. Thus, it is reasonable to focus on the numbers of periodontopathogens to prevent periodontal diseases in orthodontic patients.

In terms of sampling time, different reports mainly focused on changes of periodontal pathogens at 1 month [4], 3 months [1,20,37] or 6 months period [5] at different sites of denture following appliances removal. We set the evaluation time at 3 months post therapy when a decision needs to be made about whether apply an appropriate periodontal therapy in case of periodontal disease after appliances removal. Nevertheless, taking into account the small sample size in the present study, there is a need to further investigate clinical and microbiologic changes after orthodontic therapy with large samples.

## Conclusion

Removal of orthodontic appliances resulted in significant reduction in *P*. *intermedia*, *P*. *gingivalis* and *T*. *forsythia* in subgingival plaque associated with an obvious improvement in clinical periodontal parameters. Most clinical and microbiologic parameters tend to normal at 3 months after removal, but PD and the count of *P*. *intermedia* remained elevated compared with healthy controls, indicating that the changes induced by orthodontic therapy are only partially reversible.

## Supporting information

S1 Table The information for sample size calculation. (DOC)

S2 Table Demographic characteristics of subjects in this study. (DOC)

S3 Table Gingival index. (DOC)

S4 Table Sulcus bleeding index. The bleeding tendency of the gingival marginal was evaluated using a modified Sulcus bleeding index.
(DOC)
