# Supplementary material for: Profiling of subgingival plaque biofilm microbiota in adolescents after completion of orthodontic therapy
Source: PLoS One. 2017 Feb 3;12(2):e0171550. doi: 10.1371/journal.pone.0171550 (PMC5291508; doi:10.1371/journal.pone.0171550)
Supplement: S1 Table — (DOC) [file pone.0171550.s001.doc]

**Supplemental material**

**S1 Table. The information for sample size calculation**

| Periodontal  pathogens | Case group | | Control group | | N |
| --- | --- | --- | --- | --- | --- |
|  | mean | SD | mean | SD |  |
| Aa | 4.06 | 2.14 | 1.59 | 1.21 | 11 |
| Pi | 4.29 | 2.75 | 1.66 | 1.25 | 12 |
| Pg | 4.08 | 2.74 | 1.55 | 1.32 | 13 |
| Tf | 4.18 | 2.91 | 1.63 | 1.87 | 18 |
