# Supplementary material for: Profiling of subgingival plaque biofilm microbiota in adolescents after completion of orthodontic therapy
Source: PLoS One. 2017 Feb 3;12(2):e0171550. doi: 10.1371/journal.pone.0171550 (PMC5291508; doi:10.1371/journal.pone.0171550)
Supplement: S2 Table — (DOC) [file pone.0171550.s002.doc]

**S2 Table. Demographic characteristics of subjects in this study**

|  | Case group | Control group | *P* |
| --- | --- | --- | --- |
| subjects(n) | 20 | 19 |  |
| mean age(y)±SD | 14.42±0.86 | 14.24±0.62 | 0.46 |
| Male | 8(42.1%) | 9(47.4%) | 0.75 |
