# Supplementary material for: Profiling of subgingival plaque biofilm microbiota in adolescents after completion of orthodontic therapy
Source: PLoS One. 2017 Feb 3;12(2):e0171550. doi: 10.1371/journal.pone.0171550 (PMC5291508; doi:10.1371/journal.pone.0171550)
Supplement: S3 Table — (DOC) [file pone.0171550.s003.doc]

**S3** Table. Gingival index

| GI=0 | Normal gingival |
| --- | --- |
| GI=1 | Mild inflammation – slight change in color, slight edema. No bleeding on probing |
| GI=2 | Moderate inflammation – redness, edema, and glazing. Bleeding on probing |
| GI=3 | Severe inflammation – redness, edema, and glazing. Ulceration. Tendency to spontaneous bleeding. |
