# Supplementary material for: Profiling of subgingival plaque biofilm microbiota in adolescents after completion of orthodontic therapy
Source: PLoS One. 2017 Feb 3;12(2):e0171550. doi: 10.1371/journal.pone.0171550 (PMC5291508; doi:10.1371/journal.pone.0171550)
Supplement: S4 Table — The bleeding tendency of the gingival marginal was evaluated using a modified Sulcus bleeding index. (DOC) [file pone.0171550.s004.doc]

**S4 Table. Sulcus bleeding index. The bleeding tendency of the gingival marginal was evaluated using a modified Sulcus bleeding index**

| Score 0 | No bleeding when a periodontal probe is passed along the gingival margin. |
| --- | --- |
| Score 1 | Isolated bleeding spots visible. |
| Score 2 | Blood forms a confluent red line on margin. |
| Score 3 | Heavy or profuse bleeding. |
